# Characterization and Analysis of the Skin Microbiota in Acne: Impact of Systemic Antibiotics

**DOI:** 10.3390/jcm9010168

**Published:** 2020-01-08

**Authors:** Seo-Yeon Park, Hei Sung Kim, Se Hoon Lee, Sungjoo Kim

**Affiliations:** 1 Department of Biomedicine & Health Sciences, The Catholic University of Korea, 222 Banpo-daero, Seocho-gu, Seoul 06591, Korea; gkrud777@gmail.com; 2 Department of Dermatology, Incheon St. Mary’s Hospital, The Catholic University of Korea, Seoul 06591, Korea; leesehoon92@gmail.com

**Keywords:** acne, systemic antibiotics, impact, microbiota, microbiome, skin

## Abstract

Systemic antibiotics are extensively used to control moderate to severe acne. Hence, it is crucial to understand their impact on the skin microbiota, which is supposedly perturbed. The purpose of this study was to compare the makeup and diversity of the skin microbiota in acne patients before and after taking oral antibiotics. A longitudinal cohort study was performed on 20 participants with moderate to severe facial acne with no recent use of oral and topical antibiotics/retinoids. Patients were prescribed oral doxycycline, 100 mg, twice daily for six weeks. Skin areas on the cheek were sampled for 16S ribosomal RNA gene sequencing at baseline, and after six weeks of doxycycline treatment. Ten males and 10 females aged 11 to 44 years with a median Investigator’s Global Assessment score of 3 (moderate) were enrolled. At baseline, *Cutibacterium acnes* (formerly *Propionibacterium acnes)* was the most dominant species followed by *Staphylococcus epidermidis*. Acne severity showed a positive correlation with the abundance of *Cutibacterium acnes.* Across all subjects, antibiotic treatment reduced clinical acne grades and was associated with a 1.96-fold reduction in the relative abundance of *Cutibacterium acnes* (*p* = 0.01, 95% CI −22% to −3%). Marked changes were also identified in other bacterial species, such as *Cutibacterium granulosum* (formerly *Propionibacterium granulosum*), which increased by 4.46-fold (*p* = 0.02, 95% CI 0.004% to 0.9%) in the treated samples. In general, antibiotics administration was associated with an increase in bacterial diversity (alpha diversity). Principal coordinates analysis showed mild clustering of samples by patient (analysis of similarity, *R* = 0.135, *p* = 0.04) whereas there was scant clustering with treatment (ANOSIM, *R* = 0.005; *p* = 0.29). In conclusion, we found individuals with acne to have a unique microbial signature. Acne treatment with systemic antibiotics was associated with changes in the composition and diversity of skin microbiota, especially *Cutibacterium acnes*, which correlates with acne severity. Our study provides insight into the skin microbiota in acne and how it is modulated by systemic antibiotics.

## 1. Introduction

Acne is a chronic, inflammatory condition of the pilosebaceous unit (skin). The disease has received much attention with its high prevalence (up to 90% of teenagers are impacted, with the disease frequently continuing into adulthood), with potential for scarring leading to significant social stigma and discrimination. The pathophysiology of acne is obscure, but increased sebum production, abnormal follicular keratinization, and inflammation are thought to contribute to its occurrence. 

Colonization and triggering by *Cutibacterium acnes* (*C. acnes*; formerly called *Propionibacterium acnes*), a regular occupant of human skin and sebaceous follicles, has also been linked with acne through its impact on lipogenesis, comedone formation, and host inflammation [1,2]. The skin harbors hundreds of microorganisms, and it is highly likely that the overall balance of the bacteria on a person’s skin is as critical as *C. acnes* in acne development and skin health. While the connection between *C. acnes* and acne is well-described, far fewer studies have examined/analyzed the entire bacterial composition on acne patients’ skin [3,4,5]. 

Tetracyclines (i.e., tetracycline, doxycycline, minocycline) are often the first choice for moderate to severe acne with their ability to suppress the growth of *C. acnes* (via inhibition of protein synthesis) and control inflammation (by downregulating the production of inflammatory proteins/enzymes, such as lipase) [6]. Although useful, tetracycline antibiotics can exhibit broad-spectrum antimicrobial activity beyond *C. acnes*, having an off-target antibacterial impact on the skin microbiome. This raises concern as the commensal bacteria and its protective role in the skin may be impacted. Given the extensive use of oral tetracyclines for acne, it is necessary to understand their impact over the entire bacterial community of the skin. 

Amplification and sequencing of the 16S ribosomal RNA (rRNA) gene have been used to determine bacterial communities in various body habitats, including the skin [7]. Using this culture-free method, studies have examined the action of antibiotics in gut microbiota, often describing changes in bacterial composition following antibiotic treatment [8]. The purpose of this study was to provide an overall picture of the influence of oral antibiotics on the composition and diversity of the acne skin microbiota, which is supposedly altered. 

## 2. Materials and Methods

### 2.1. Study Design

Patients newly diagnosed with acne by a dermatologist were enrolled in August 2017 to June 2018, at the Department of Dermatology, Incheon St. Mary’s Hospital, Korea. Inclusion criteria were moderate to severe acne (grades 3 and 4 on the Investigator’s Global Assessment (IGA) grading scale) with the presence of many comedonal and inflammatory acne on the face, and willingness to avoid facial washing and application of topical agents to the face for 12 h prior to skin sampling. Exclusion criteria included a history of systemic or topical antibiotic use within one month of the baseline study visit, hypersensitivity to tetracyclines, systemic acne treatment within four weeks, topical acne treatment within two weeks, significant facial hair interfering with sampling, pregnancy or breast feeding status, and inability to provide an informed consent. The study was approved by Incheon St. Mary’s Hospital (The Catholic University of Korea) Institutional Review Board (OC17TNSI0057), and participants provided written informed consent prior to participation.

### 2.2. Antibiotic Treatment and Sample Collection

Participants were instructed to take doxycycline, 100 mg, twice daily for six weeks. Skin samples were collected at two visits across six weeks, one before treatment initiation and another approximately six weeks after starting doxycycline therapy. Participants’ compliance to antibiotic treatment was checked at a separate visit made two weeks after the start of doxycycline. Here, the possible side effects of doxycycline, such as nausea and diarrhea, were inquired. Afterwards, all participants were asked to write a self-reported diary on the medication. At each visit (at baseline and after six weeks of doxycycline), skin samples were collected from the cheeks (a single sample covering both cheeks, 4 cm^2^ area per side) using sterile cotton swabs (EASY SWAB, Hanil-Komed Inc., Seongnam, Gyeonggi-do, Korea). Each side was rubbed 20 times with the cotton stick: 10 times in one direction and 10 times perpendicular to this direction. Microbiota sampling was conducted by the same investigator (H.S.K.) at all study visits. 

### 2.3. DNA Extraction and 16S rRNA Gene Polymerase Chain Reaction Amplification and Sequencing

DNA was separated from the skin samples using an enzymatic lysis and bead-based tissue homogenization protocol; the samples were incubated shortly in a lytic enzyme mixture of lysozyme, mutanolysin, proteinase K, and lysostaphin, followed by mechanical lysis with silica beads (0.1 mm), as published beforehand [9]. The DNA cleanup was then conducted with a fecal DNA extraction kit (ZR Fecal DNA MiniPrep; Zymo Research). After DNA extraction, the V3–V4 hypervariable region of the 16S rRNA gene was amplified by polymerase chain reaction and sequenced using the Illumina HiSeq platform (250 base pairs, paired-end reads) as reported previously [10,11].

### 2.4. Data Analysis

After sequencing, de-multiplexing of the data based on the Illumina index reads was performed and the raw data were converted to FASTQ files. Illumina adapters were removed using the FASTP program [12] and error correction was performed on the region where the two reads overlapped. The paired reads were merged using FLASH v1.2.11 [13]. For precise operational taxonomic units (OTUs) analysis, data containing sequence error (i.e., merged sequences shorter than 400 bp, raw reads with ambiguous base cells, chimeric sequences) were removed. The remaining representative reads from non-chimeric clusters were clustered de novo into OTUs (97% similarity threshold) using a CD-HIT-EST-based OTU analysis program (CD-HIT-OTU) (CD-HIT stands for Cluster Database at High Identity with Tolerance. CD-HIT is a clustering program developed by Weizhong Li’s lab at the University of California, San Diego. CD-HIT-EST is one of the programs in the CD-HIT package) [14]. Afterwards, taxonomic assignments were performed using the basic local alignment search tool (BLASTN v2.4.0, http://blast.ncbi.nlm.nih.gov/Blast.cgi) [15] and the reference database (National Center for Biotechnology Information 16S, Bethesda, MD, USA). To improve the accuracy of taxonomic assignments, a top BLAST hit with alignment that spanned <85% of the original query sequence and a top BLAST hit with a percent identity less than 85% were not assigned. Observed relative abundances were estimated by dividing the observed number of 16S rRNA amplicon reads by the total number of reads per sample. Microbiota α diversity, representing the microbial diversity within an individual sample, was computed in QIIME v1.9 [16] through the whole tree phylogenetic diversity metric. Microbiota β diversity, which indicates the inter-variability of microbial diversity between samples, was examined through principal coordinates analysis of weighted UniFrac distances in QIIME and hierarchical clustering based on the unweighted pair group method with the arithmetic mean algorithm in the R statistical software (R Core Team). The flexible relationship between the samples were visualized through the PCoA and UPGMA tree. 

### 2.5. Statistical Analysis

Comparison of α diversity and the relative abundance of bacterial taxa between samples (complete sample sets from pre- and post-treatment groups), sample sets taken before the initiation of treatment based on age (under 20 vs. over 20), sex (male vs. female), and acne severity (IGA 3 vs. IGA 4) were performed with Wilcoxon signed rank test/Wilcoxon rank sum test in R package v3.0.1. (http://cran.r-project.org/web/packages/gPCA/index/html). All other analyses and visualizations were performed with R and the boxplot package. Permutation tests were used to calculate statistical differences in microbiota in PCoA. For all statistical analyses, two-sided *p* < 0.05 was statistically significant. 

## 3. Results

Demographics and relevant clinical features of the 20 patients included in this study are shown in Table 1 and Table S1. Our study included 20 Asian subjects with skin phototype 3 (25%), 4 (70%), and 5 (5%) on the Fitzpatrick scale. Half of the subjects (50%) were female; the mean age was 19.6 ± 7.5 years. The mean acne severity (IGA) at baseline was 3 ± 0.4; on average, at day 0, subjects had 15.2 ± 6.7 papulo-pustular lesions on the entire face. After six weeks of oral doxycycline, the mean acne severity (IGA) was measured as 1.9 ± 0.6.

### 3.1. Taxonomic Assignment

**Figure 1:**
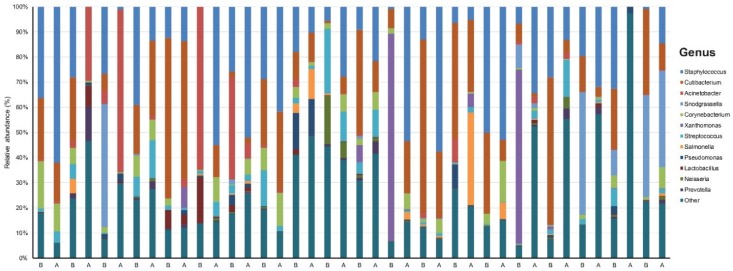
Taxonomy plot of the microbial communities of acne patients before and after six weeks of doxycycline.

Our data set involved 40 samples across 20 patients sequenced to a mean (SD) read count of 151,064 (27,070) (Table S2). We identified 19 phyla, 27 classes, 75 orders, 181 families, 590 genera, and 1716 species that were unique and present in at least one sample. There was a dominance of *Staphylococcus* (23.8%) and *Cutibacterium* (19.9%) at the genus level across all samples (Figure 1). 

### 3.2. Relative Abundance of Individual Bacterial Taxa Pre- and Post-Doxycycline Treatment

**Figure 2:**
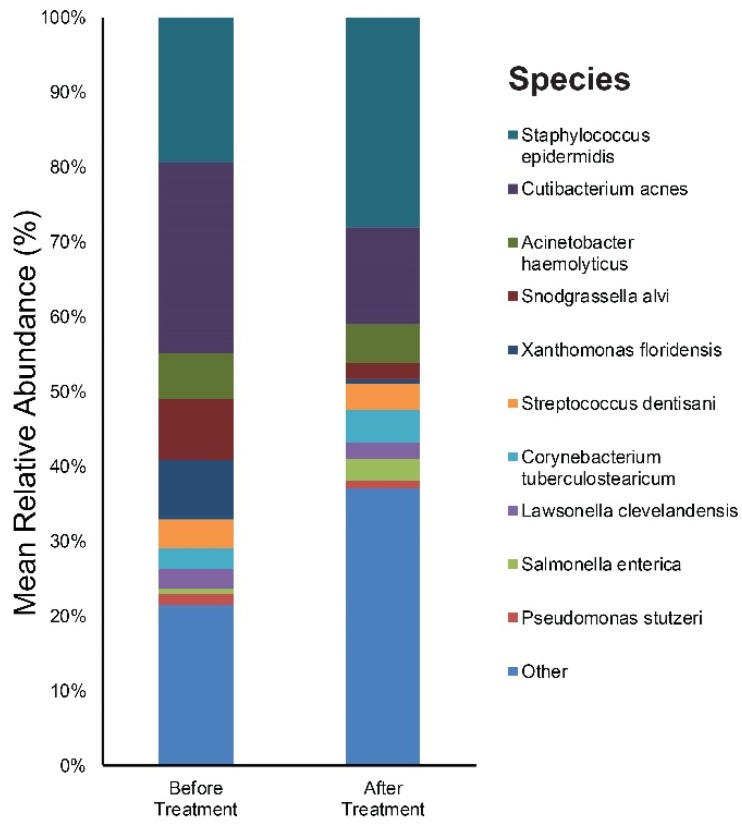
Bar graph on skin microbiota in acne patients before and after six weeks of doxycycline.

We focused on the species level when assessing changes in the abundance of individual bacterial taxa relative to the entire bacterial community in samples. Figure S1A,B provide results of the main bacterial phyla, genus, and species in our 20 subjects at baseline and after six weeks of doxycycline, respectively. *Cutibacterium* (26%), *Staphylococcus* (20%), and *Snodgrassella* (8%) were the main bacterial genera found in untreated acne skin. The most dominant taxonomic groups at the species level in acne skin were: *C. acnes* (25%) followed by *Staphylococcus epidermidis* (*S. epidermidis*) (19%), and *Snodgrassella alvi* (8%) (Figure 2). After six weeks of oral antibiotics, the predominant genera were as follows: *Staphylococcus* (29%), *Cutibacterium* (14%), and *Acinetobacter* (6%). Among the bacterial species, *S. epidermidis* (28%) was most commonly found in the skin samples followed by *C. acnes* (13%), *Actinetobactor haemolyticus* (5%), *Corynebacterium tuberculostearicum* (4%), *Snodgrassella alvi* (2%), and *Cutibacterium granulosum* (1%) (Figure 2). 

**Figure 3:**
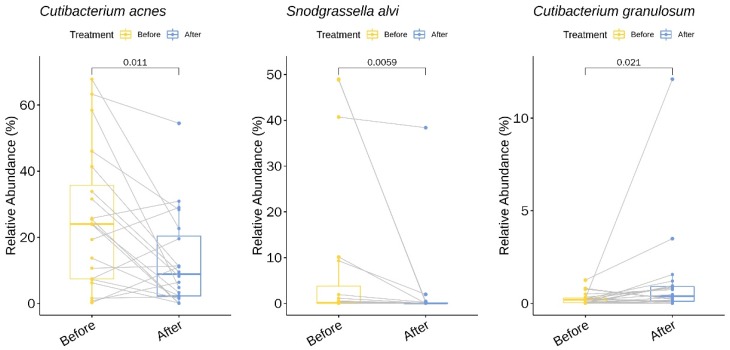
Bacterial species with a significant change in the relative abundance (%) upon doxycycline treatment.

We identified two genera (with a relative abundance greater than 0.1% across all samples) and three species with statistically significant changes in relative abundance upon treatment with doxycycline (Table S3 and Figure 3). Among the three species, two showed a decrease in the mean relative abundance: *C. acnes* decreased 1.96-fold from baseline (pre-treatment) to week six (post-treatment) (*p* = 0.01, 95% CI −22% to −3%) and *Snodgrassella alvi* decreased 3.85-fold (*p* < 0.01, 95% CI −24% to −0.2%). In contrast, one species showed an increase in the relative abundance following treatment with doxycycline: *Cutibacterium granulosum* increased 4.46-fold (*p* = 0.02, 95% CI 0.04% to 0.9%).

### 3.3. Relative Abundance of Individual Bacterial Taxa at Baseline According to Age, Sex, and Acne Severity

**Figure 4:**
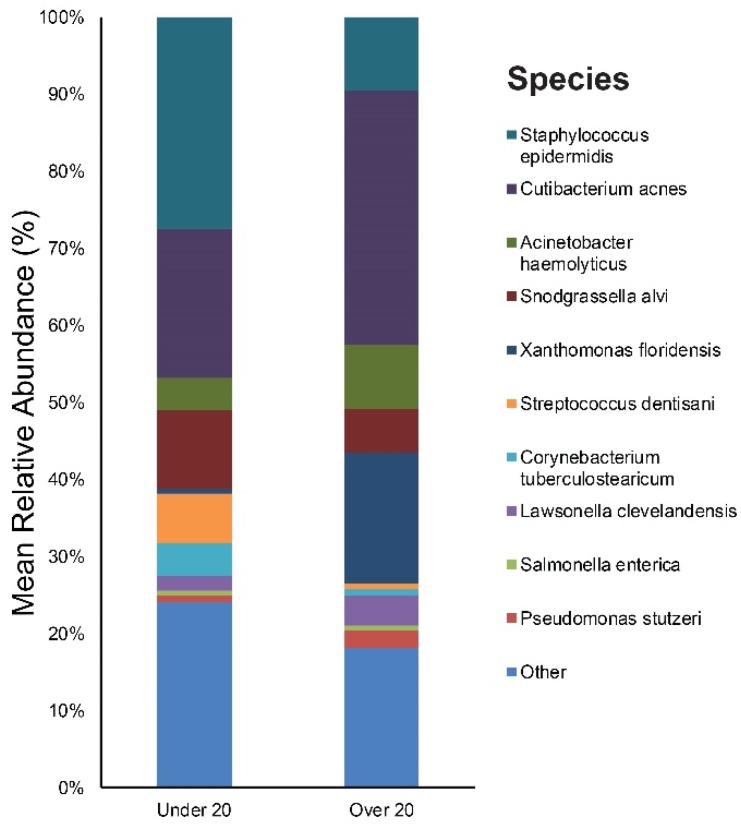
Bar graph on baseline skin microbiota in acne patients according to age (under 20 and over 20).

The study participants were divided into two age groups: The children/adolescent group (aged under 20 years, *n* = 11, mean age: 14), and the young adult group (aged over 20 years, *n* = 9, mean age: 26). Figure S2 provides the results of the main bacterial phyla, genus, and species in our under 20, and over 20 subjects, respectively. *Staphylococcus* (28%), *Cutibacterium* (20%), and *Snodgrassella* (10%) were the main bacterial genera found in the under 20 age group’s skin. The most dominant taxonomic groups at the species level in under 20 skin were: *S. epidermidis* (27%), followed by *C. acnes* (19%) and *Snodgrassella alvi* (10%) (Figure 4). In the over 20 age group, the predominant genera were as follows: *Cutibacterium* (33%), *Xanthomonas* (17%), and *Staphylococcus* (10%). Among the bacterial species, *C. acnes* (33%) was most commonly found in the skin samples followed by *Xanthomonas floridensis* (17%) and *S. epidermidis* (10%) (Figure 4). 

**Figure 5:**
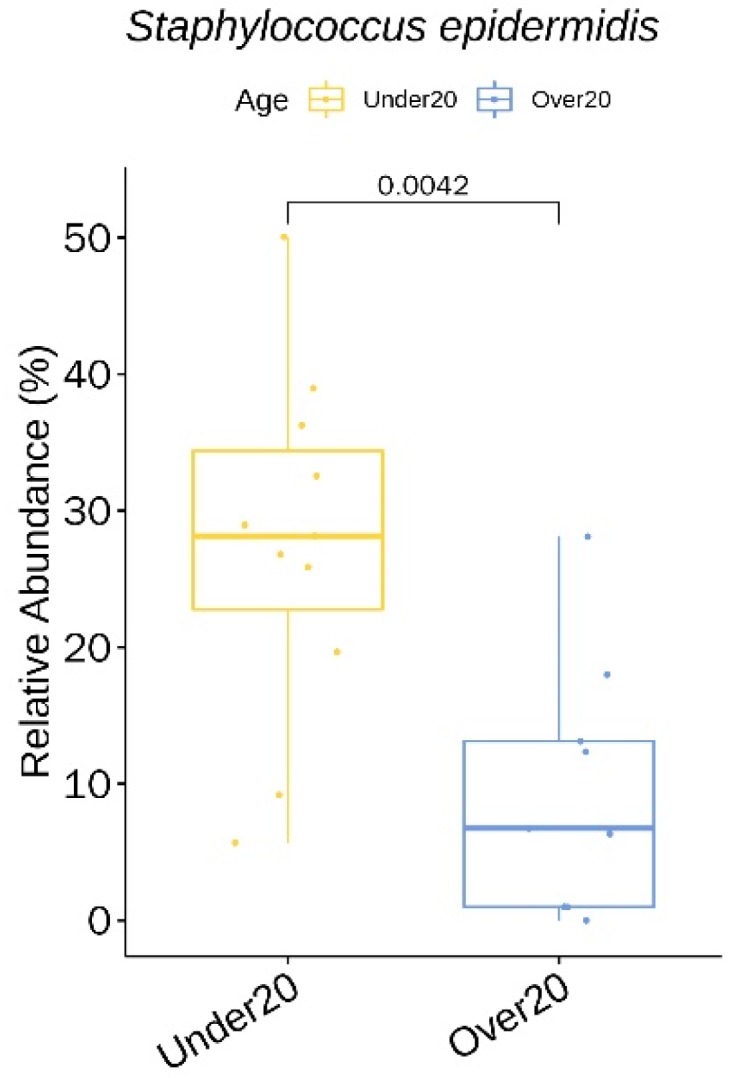
*Staphylococcus epidermidis* showing a significantly higher relative abundance in the under 20 age group at baseline.

We identified three genera (with a relative abundance greater than 0.1% across all samples) and seven species with a statistically significant difference in the relative abundance between the two age groups (Table S4). All seven species showed a higher relative abundance in the under 20 age group: *Staphylococcus epidermidis* (Figure 5), *Corynebacterium matruchotii*, *Corynebacterium durum*, *Corynebacterium tuberculostearicum*, *Streptococcus thermophilus*, *Streptococcus dentisani*, and *Corynebacterium timonense*.

**Figure 6:**
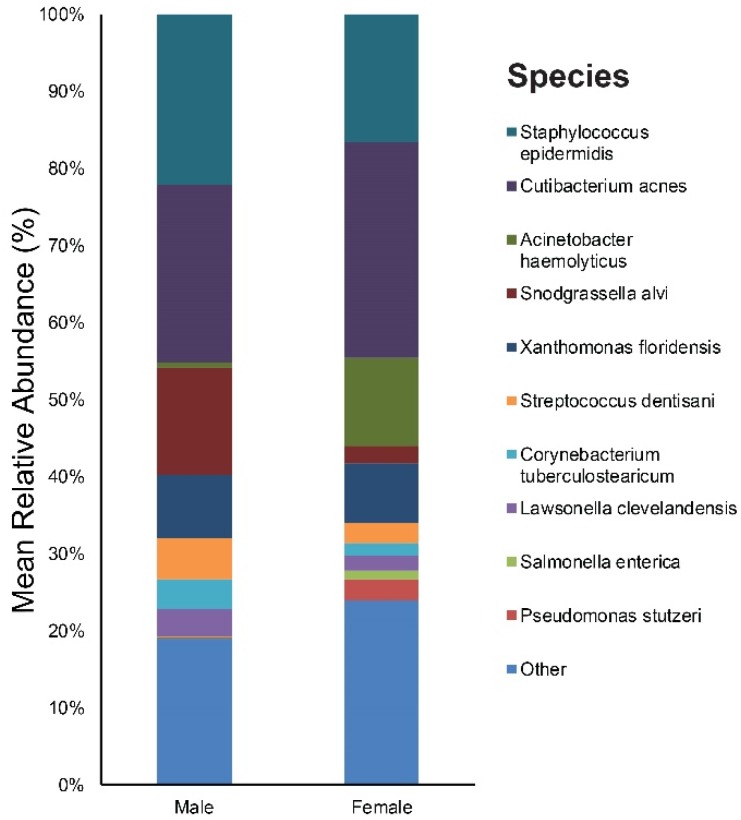
Bar graph on baseline skin microbiota according to sex (male and female).

As for sex, our study included 10 males and 10 females. Figure S3 provides the results of the main bacterial phyla, genus, and species in male and female subjects, respectively. *Cutibacterium* (23%), *Staphylococcus* (22%), and *Snodgrassella* (14%) were the main bacterial genera found in males. The most dominant taxonomic groups at the species level in male skin were *C. acnes* (23%) followed by *S. epidermidis* (22%) and *Snodgrassella alvi* (14%) (Figure 6). In the female group, the predominant genera were as follows: *Cutibacterium* (28%), *Staphylococcus* (17%), and *Acinetobacter* (12%). Among the bacterial species, *C. acnes* (28%) was most commonly found in the skin samples followed by *S. epidermidis* (17%), *Acinetobacter haemolyticus* (12%), and *Xanthomonas floridensis* (8%) (Figure 6). 

We identified one genus (with a relative abundance greater than 0.1% across all samples) and one species with a significant difference in the relative abundance between the male and female group (Table S5). *Pseudomonas putida* showed a higher relative abundance in females. 

**Figure 7:**
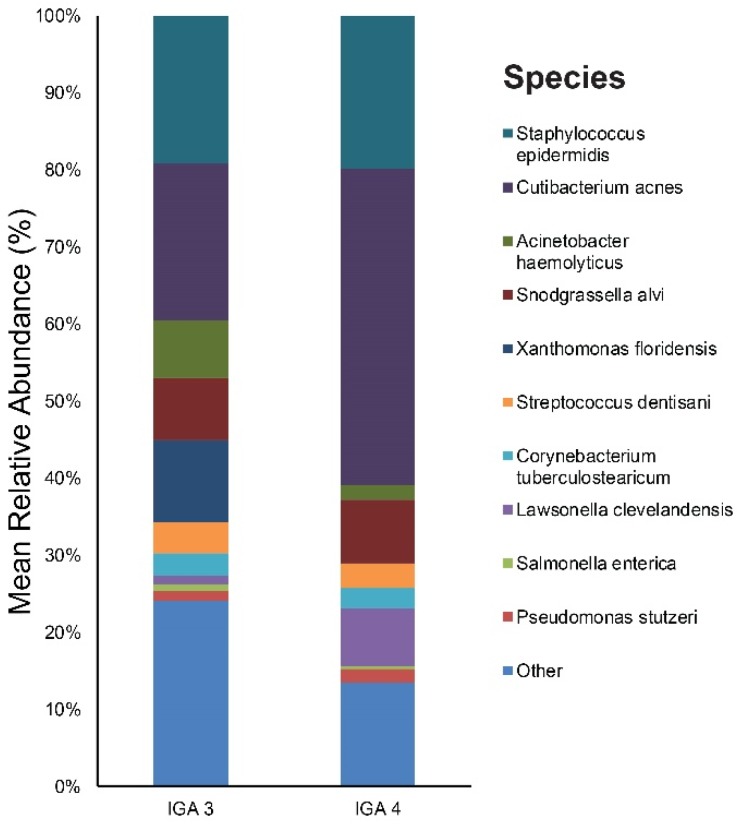
Bar graph on baseline skin microbiota according to acne severity (IGA 3 and IGA 4), IGA, Investigator’s Global Assessment.

In terms of acne severity (IGA), 15 patients were IGA grade 3 and 5 were IGA grade 4. Figure S4 provides the results of the main bacterial phyla, genus, and species in our IGA grade 3 and IGA grade 4 subjects, respectively. *Cutibacterium* (21%), *Staphylococcus* (19%), and *Xanthomonas* (11%) were the main bacterial genera found in the IGA 3 group’s skin. The most dominant taxonomic groups at the species level in IGA 3 skin were: *C. acnes* (20%), followed by *S. epidermidis* (19%), *Xanthomonas floridensis* (11%), and *Snodgrassella alvi* (8%) (Figure 7). In the IGA 4 group, the predominant genera were as follows: *Cutibacterium* (42%), *Staphylococcus* (21%), and *Snodgrassella* (8%). Among the bacterial species, *C. acnes* (41%) was most commonly found in the skin samples followed by *S. epidermidis* (20%) and *Snodgrassella alvi* (8%) (Figure 7). 

**Figure 8:**
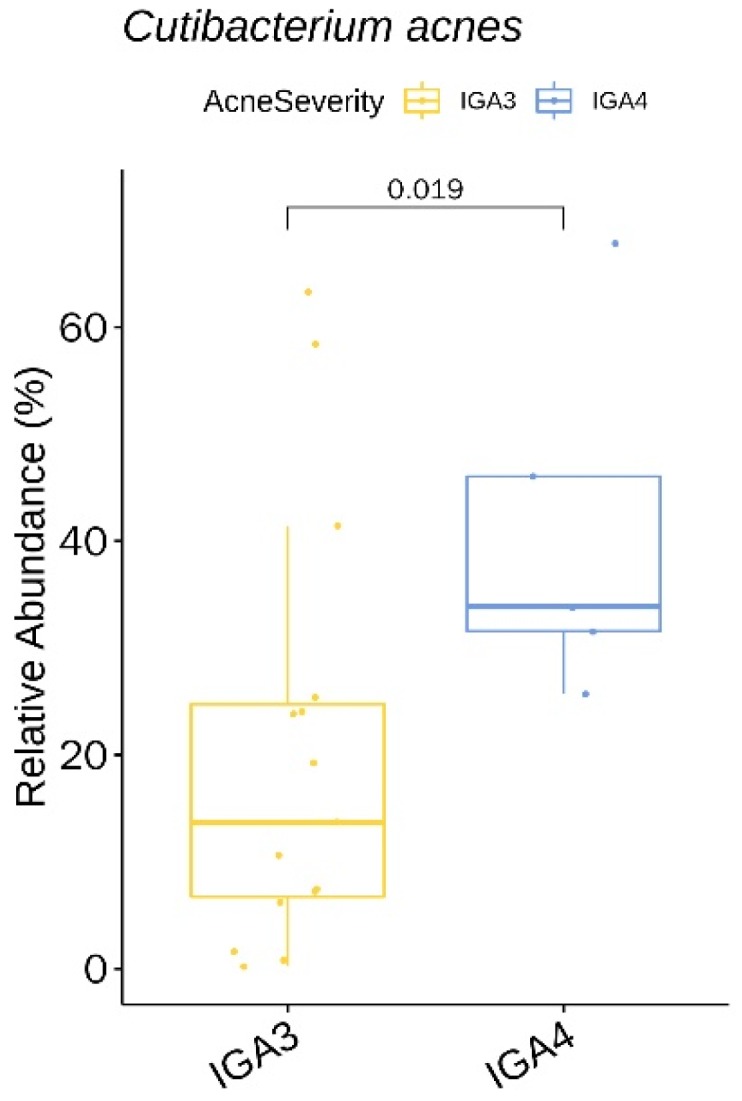
*Cutibacterium acnes* showing a significantly lower relative abundance in the IGA 4 acne severity group at baseline.

We identified two genera (with a relative abundance greater than 0.1% across all samples) and two species with a significant difference in the relative abundance between acne severity (IGA) 3 and the IGA 4 group (Table S6). The two species, *Cutibacterium acnes* (Figure 8) and *Lawsonella clevelandensis*, showed a higher relative abundance in the IGA 4 group.

### 3.4. α Diversity

**Figure 9:**
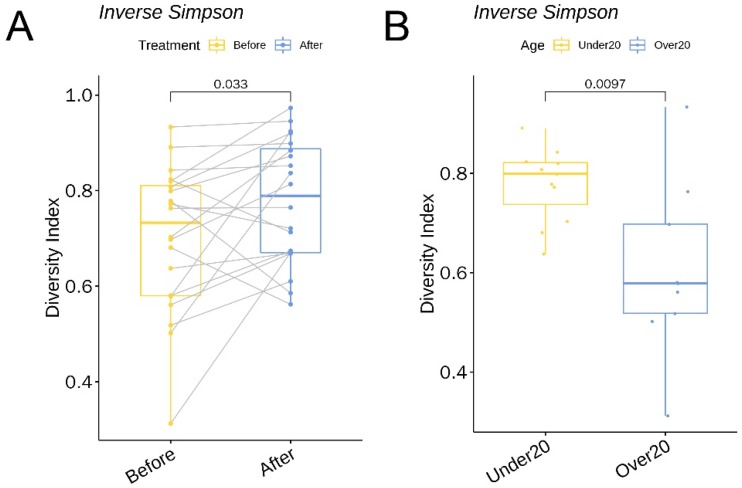
A boxplot of the inverse Simpson index (**A**) comparing skin microbiota before and after treatment and (**B**) comparing baseline skin microbiota in patients under 20 and over 20 years.

The *α* diversity between the before-treatment and after-treatment group was compared using the inverse Simpson and Shannon indices. The Shannon index increased 1.27-fold (*p* = 0.03, 95% CI 0.1 to 1.5) and the inverse Simpson increased 1.11-fold (*p* = 0.03, 95% CI 0.005 to 0.14) (Figure 9A) following six weeks of oral doxycycline.

As for the *α* diversity between the under 20 and over 20 age group, the inverse Simpson was 1.29-fold (*p* = 0.01, 95% CI 0.06 to 0.3) higher in the under 20 subjects (Figure 9B).

### 3.5. β Diversity

**Figure 10:**
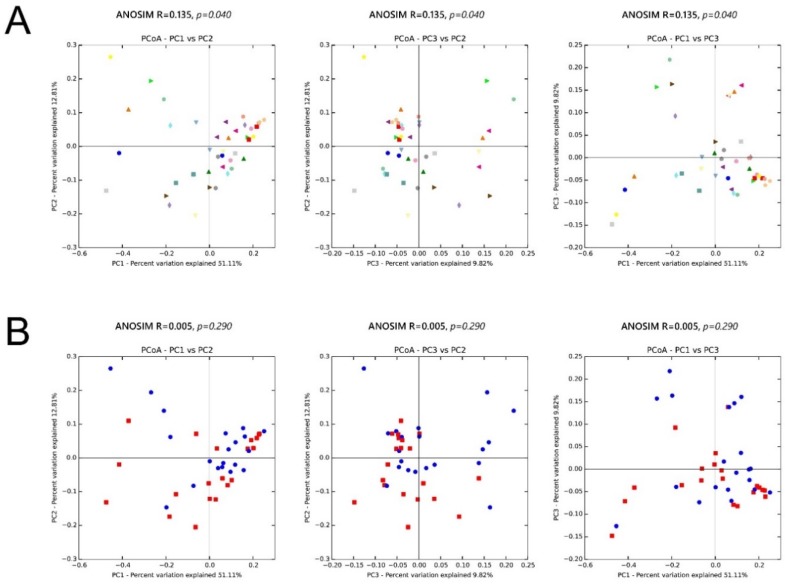
Microbiota β diversity (between-sample microbial diversity) based on principal coordinate analysis (PCoA) of weighted UniFrac distances. Two-dimensional PCoA plots display inter-sample distances by three principal coordinates (PC1, PC2, and PC3) with labeling of individual samples by (**A**) patient and (**B**) treatment, for patients 1 to 20. Principal coordinates, calculated from a distance matrix of weighted Unifrac distances, have no units.

We also assessed the inter-sample diversity, or *β* diversity, based on principal coordinates analyses of weighted UniFrac distances. Similarity between samples across the three principal coordinates (PC1, PC2, and PC3) with samples that cluster close to one another indicates a similar bacterial composition between those samples. The ANOSIM (Analysis of Similarity), which generates an *R* test statistic stretching from −1 to 1, was used to measure the clustering of samples by patient and treatment (Figure 10). A positive *R* value indicates greater within-group similarity than between-group similarity, with greater magnitudes of the *R* value suggesting stronger clustering of samples. An *R* value of 0 indicates no clustering of samples, whereas a negative *R* value suggests greater between-group resemblance than within-group similarity. Although there was mild clustering of samples by patient (ANOSIM, *R* = 0.135; *p* = 0.04), showing the distinctive microbial signature for each person, clustering by treatment (ANOSIM, *R* = 0.005; *p* = 0.29) was much less apparent. 

## 4. Discussion

The role of the skin microbiome in acne is of great interest to clinicians, patients, and researchers. This interest originates in part from the long-established view that *C. acnes* has a causative role in acne pathogenesis [2,17] and the efficacy of oral and topical antibiotics in acne treatment. Our longitudinal cohort study examined the skin microbiota of untreated acne skin and the influence of systemic antibiotics on acne skin microbiota. Through 16sRNA gene sequencing, we found *C. acnes* and *S. epidermidis* to be prevalent in the untreated skin samples, consistent with prior findings [3,4,5]. Present in healthy and diseased skin, their roles as commensals or opportunistic organisms are not completely understood.

Cyclines are broad-spectrum bacteriostatic antibiotics and are the most commonly prescribed oral antibiotics for moderate to severe inflammatory acne [18]. Tetracyclines have multiple mechanisms of action, including an antibacterial effect, inhibition of pro-inflammatory mediators and tissue destructive enzymes, and modulation of innate immunity, but it is not known which mechanism is the most relevant in acne patients [19]. As for our study, treatment with doxycycline led to clinical improvements in acne severity concurrent with a 1.96-fold reduction of *C. acnes*. 

Our findings of significant changes in the relative abundance (%) of multiple bacterial species may also have important clinical implications. In addition to the reduction of *C. acnes,* we observed a 3.85-fold decrease in *Snodgrassella alvi* (*S. alvi*) after six weeks of doxycycline treatment. *S. alvi,* a species known as a gut symbiont of bees, has been identified as a core microbiota of *Demodex* mites from rosacea patients [20] and was also prevalent (8%) in our untreated skin samples. Interestingly, an association between *Demodex* infestation and acne has been reported in prior studies [21,22,23]. Although our findings suggest a subtle link between acne and *Demodex* infestation, there is some doubt on the origin of *S. alvi.* Since *Demodex* mites usually reside in the hair follicles and sebaceous glands, they would not have been thoroughly collected through skin swabbing.

*Cutibacterium granulosum (C. granulosum)* increased 4.46-fold. As part of the skin microbiota, *C. granulosum* is found in sebum-rich areas but at a much lower abundance than *C. acnes.* Since *C. granulosum* co-localizes with *C. acnes* in the stratum corneum and hair follicles [24], there is a possibility that *C. acnes* competes with *C. granulosum* for efficient acquisition of nutrients from host components. The skin microbiota of papulopustular rosacea patients was reported to be depleted in *C. granulosum* [25], which suggests that this species may play a role in maintaining the normal skin barrier by averting the growth of potential pathogens. 

*S. epidermidis* was the second most prevalent microbiota (19%) before treatment and became the most prevalent bacteria (28%) after treatment. *S. epidermidis* and *C. acnes* use glycerol as a shared carbon source to generate different short-chain fatty acids used as antimicrobial agents to compete against each other [26]. Different in vivo antagonism investigations demonstrated that *S. epidermidis* controls the proliferation of *C. acnes* via the release of succinic acid, a fatty acid fermentation product, which blocks surface toll-like receptors (TLRs) of keratinocytes and tumor necrosis factor and suppresses *C. acnes*-induced IL-6 [27,28,29]. We think our findings of increased *S. epidermidis* concomitant with clinical improvement of acne and decreased *C. acnes* add further evidence that *C. acnes* and *S. epidermidis* interact. Application of *S. epidermidis*-encapsulated polysulfone microtube array membranes plus glycerol onto the *C. acnes*-injected mouse ears considerably lessened the growth of *C. acnes* and the production of macrophage inflammatory protein-2 [30], implicating the potential use of live *S. epidermidis* in acne probiotics.

The skin microbiota of our study patients became significantly more diverse after exposure to doxycycline. The increase in alpha diversity (within-sample microbial diversity) is likely from reduced *C. acnes* colonization, which encourages the growth of other flora by freeing up niche space. As for *β* diversity, which is the inter-sample diversity, there was mild clustering of samples by patient (ANOSIM, *R* = 0.135; *p* = 0.04), indicating a distinctive microbial signature for each person. Conversely, clustering by treatment (ANOSIM, *R* = 0.005; *p* = 0.29) was much less apparent, consistent with data showing that the skin microbiome is highly individualized and that specific bacterial taxa may persist on a given person for months to years. The finding emphasizes the importance of collecting paired samples from the same patient. 

Age is known to influence the skin microbiota composition. The relative abundance of *C. acnes* in our under 20 age group (mean age: 14) was lower than that of the over 20 age group (mean age: 26) (19% vs. 33%), which is compatible with the study results by Zhai et al. [31]. In this particular study, *C. acnes* evolved with age, showing the lowest abundance in childhood (four to six years), a dramatic increase at puberty (11–13 years), a peak in young adults (25–34 years), and a subsequent decline with age. The abundance of *C. acnes* in young adults may be explained by the fact that sebaceous glands fully mature and reach the highest level of secretion in late puberty/early adulthood. *C. acnes* inhibits *Staphylococcus*, which explains the statistically higher relative abundance of *S. epidermidis* in the under 20 population. The alpha diversity of the under 20 age group (11–18 years) was higher than that of the over 20 age group (22–44 years). This is again supported by the results from Zhai et al. [31], where the elderly (62–74 years), children, and adolescents exhibited more species richness and diversity than the young and middle-aged adults (37–53 years). 

The influence of sex on the composition and diversity of the bacterial communities was relatively small compared to age. Still, we found a higher relative abundance of *Pseudomonas* in females, which is in line with findings from a prior study [31]. This variation is thought to rise from the difference in sebum levels as well as the habitual use of cosmetics and moisturizers among the female population. 

As for acne severity, the prevalence of *C. acnes* correlated with the severity of acne, which suggests that it is at the center of the disease process. Several mechanisms have been suggested by which *C. acnes* aggravates acne, including comedone formation, augmentation of lipogenesis, and host inflammation [2,17].

A potential limitation of our study is the use of swabs for skin sampling, which may fail to capture the bacterial community of the pilosebaceous unit. Although a recent study by Hall et al. [32] showed no difference in *C. acnes*-associated factors between surface and follicular sampling methods, simultaneous examination of the follicular microbiota may add insights on shifts in the skin microbiota observed in this study. Future iterations of our study may also use negative and mock community controls to account for foreign DNA that may have been brought into the 16S rRNA gene analysis. Synthetic fiber swabs can be used as an alternative to cotton swabs, which is a potential source of DNA and a confounder to the results. 

We used primers targeting the V3–V4 hypervariable region of the 16S rRNA gene, which identified significant levels of *Cutibactierum* and *C. acnes* compared to studies that singly targeted the V4 region [4]. However, the obtained relative abundance of *Cutibacterium* was lower than that reported in previous studies targeting the V1–V3 region [32]. 

Our study did not include a further follow-up, but it would be interesting to explore what happens to the skin microbiota in acne after completion of the treatment course, and how quickly the bacterial communities return to their initial state.

Finally, we were unable to obtain strain-level resolution of *C. acnes.* Considering the emerging association of type IA1 strains of *C. acnes* with inflammatory skin disease [33], it would be valuable to identify the *C. acnes* strains in acne and examine their alteration with antibiotic treatment. 

## 5. Conclusions

Our findings suggest that *C. acnes* plays an active role in Asian acne patients, with its dominance in untreated skin, reduction following the use of antibiotics (with concurrent clinical improvement), and its correlation with acne severity. Also, we showed that the interplay between *C. acnes* and other bacteria, for example, *S. epidermidis*, exists at all times (untreated skin vs. with antibiotics use) and is integral for skin health. Deeper understanding of changes in the function of the bacterial species/strains, past density, and diversity will be needed to define their precise role and hence, possible intervention points in acne pathogenesis and treatment. 

## Figures and Tables

**Table 1:** Demographic and clinical characteristics of the study participants.

General Characteristics (*n* = 20)	
Sex (M/F), *n* (%)	10/10 (50%)
Age (years), median (range)	18 (11–44)
Fitzpatrick Skin Type, median (range)	4 (3–5)
Duration of acne (years), median (range)	2 (less than a year—10)
Baseline Acne severity (IGA), median (range)	3 (3–4)
Acne severity (IGA) after 6 weeks of oral doxycycline, median (range)	2 (1–3)
Baseline Inflamed Lesion Count, median (range)	15 (6–30)

## Supplementary Materials

The following are available online at https://www.mdpi.com/2077-0383/9/1/168/s1, Figure S1: Krona graph on skin microbiota in acne patients (A) Before treatment, and (B) After 6 weeks of doxycycline; Figure S2: Krona graph on baseline skin microbiota in acne patients (A) Under 20, and (B) Over 20 years of age; Figure S3: Krona graph on baseline skin microbiota in (A) Male, and (B) Female acne patients; Figure S4: Krona graph on baseline skin microbiota according to acne severity. (A) IGA 3, and (B) IGA 4; Table S1: Demographic and Clinical Characteristics of the Study Participants in detail; Table S2: Sample read counts; Table S3: Bacterial genera (with relative abundance of greater than 0.1% across all samples) and species with significantly higher mean relative abundance (A) before and (B) after doxycycline treatment; Table S4: Bacterial genera (with relative abundance of greater than 0.1% across all samples) and species with significant difference in relative abundance between the two age groups (Under 20, Over 20) at baseline. All showed a higher relative abundance in the Under 20 age group; Table S5: Bacterial genera (with relative abundance of greater than 0.1% across all samples) and species with significant difference in relative abundance between the Male and Female group at baseline. Pseudomonas showed a higher relative abundance in Female subjects; Table S6: Bacterial genera (with relative abundance of greater than 0.1% across all samples) and species with significant difference in relative abundance between acne severity (IGA) 3 and IGA 4 group at baseline. All showed a higher relative abundance in the IGA 4 subjects.
