# Supplementary material for: Characterization and Analysis of the Skin Microbiota in Acne: Impact of Systemic Antibiotics
Source: J Clin Med. 2020 Jan 8;9(1):168. doi: 10.3390/jcm9010168 (PMC7019264; doi:10.3390/jcm9010168)
Supplement: Supplementary file 1 [file jcm-09-00168-s001.pdf]

Table S1. Demographic and Clinical Characteristics of the Study Participants in detail.

No.	Sex	Age	Race	IGA score (before)	IGA score (after)	Prior treatment at baseline	Use of lotions and makeup*
1	M	14	Asian	3	2	None	None/ None
2	F	14	Asian	3	1	None	None/ Lotion
3	M	14	Asian	3	1	None	Lotion/ None
4	M	13	Asian	3	2	None	None/ None
5	F	26	Asian	3	2	Oral doxy last taken 3 months ago	None/ None
6	F	23	Asian	3	1	None	None/ None
7	F	13	Asian	3	2	None	None/ None
8	M	18	Asian	4	2	Oral doxy last taken 7 months ago	None/ None
9	F	25	Asian	3	1	Oral roxithromycin last taken 3 years ago	None/ None
10	M	11	Asian	3	2	None	None/ None
11	F	12	Asian	3	2	None	None/ None
12	M	23	Asian	3	2	Oral doxy last taken 2 months ago	None/ None
13	M	25	Asian	4	2	Oral doxy last taken 5 years ago	None/ None
14	F	22	Asian	4	3	Oral doxy last taken 6 months ago	Lotion/ None
15	F	18	Asian	4	3	None	None/ None
16	M	44	Asian	3	2	Oral doxy last taken 4 months ago	None/ None
17	F	24	Asian	3	2	Oral doxy last taken 2 years ago	None/ None
18	M	15	Asian	3	2	None	None/ None
19	F	15	Asian	3	2	Oral doxy last taken 1 year ago	None/ None
20	M	23	Asian	4	2	None	Lotion/ None

*Use of lotion and makeup on the skin the day of sampling (baseline, after doxycycline) noted by subject

Table S2. Sample read counts.

Sample No	Before/ After Doxycycline	Read Count (CD-HIT-OUT)
1	Before	164,611
	After	204,225
2	Before	127,667
	After	141,707
3	Before	181,178
	After	148,717
4	Before	155,291
	After	136,972
5	Before	146,360
	After	117,788
6	Before	169,996
	After	129,478
7	Before	159,777
	After	166,527
8	Before	162,885
	After	157,468
9	Before	150,501
	After	136,144
10	Before	149,869
	After	163,871
11	Before	122,086
	After	163,483
12	Before	177,030
	After	140,143
13	Before	151,230
	After	173,296
14	Before	106,806
	After	157,419
15	Before	135,024
	After	175,975
16	Before	172,405
	After	143,355
17	Before	136,349
	After	135,118
18	Before	192,559
	After	153,847
19	Before	150,898
	After	36,149
20	Before	128,910
	After	133,329

Table S3. Bacterial genera (with relative abundance of greater than 0.1% across all samples) and species with significantly higher mean relative abundance (A) before and (B) after doxycycline treatment.

A

Genera	Fold-increase	*P* value	95% CI
Cutibacterium	1.82	0.02	2.3% - 21%
Snodgrassella	3.85	0.006	0.2% - 24%

Species	Fold-increase	*P* value	95% CI
Cutibacterium acnes	1.96	0.02	3% - 22%
Snodgrassella alvi	3.85	0.006	0.2% – 24%

B

Species	Fold-increase	*P* value	95% CI
Cutibacterium granulosum	4.46	0.02	0.04% - 0.9%

Table S4. Bacterial genera (with relative abundance of greater than 0.1% across all samples) and species with significant difference in relative abundance between the two age groups (Under 20, Over 20) at baseline. All showed a higher relative abundance in the Under 20 age group.

Genera	Fold-increase	*P* value	95% CI
Staphylococcus	2.85	0.004	7.3% - 28%
Corynebacterium	3.77	0.01	0.55% - 6.25%
Streptococcus	6.58	0.01	0.4% - 6.89%
**Species**	**Fold-increase**	***P* value**	**95% CI**
Staphylococcus epidermidis	2.85	0.004	7.3% - 28%
Corynebacterium matruchotii	76	0.002	0.02% - 0.71%
Corynebacterium durum	16	0.004	0.006% - 0.14%
Corynebacterium tuberculostearicum	4.89	0.006	0.44% - 4.59%
Streptococcus thermophilus	5.49	0.008	0.01% - 0.27%
Streptococcus dentisani	7.63	0.01	0.41% - 6.51%
Corynebacterium timonense	4.59	0.0497	0.00003% - 0.15%

Table S5. Bacterial genera (with relative abundance of greater than 0.1% across all samples) and species with significant difference in relative abundance between the Male and Female group at baseline. *Pseudomonas* showed a higher relative abundance in Female subjects.

Genera	Fold-increase	*P* value	95% CI
Pseudomonas	12	0.007	0.07% - 4.08%
**Species**	**Fold-increase**	***P* value**	**95% CI**
Pseudomonas putida	34	0.0002	0.04% - 0.15%

Table S6. Bacterial genera (with relative abundance of greater than 0.1% across all samples) and species with significant difference in relative abundance between acne severity (IGA) 3 and IGA 4 group at baseline. All showed a higher relative abundance in the IGA 4 subjects.

Genera	Fold-increase	*P* value	95% CI
Cutibacterium	2.03	0.02	4.53% - 44%
Lawsonella	6.44	0.0009	1.49% - 11%
**Species**	**Fold-increase**	***P* value**	**95% CI**
Cutibacterium acnes	2.02	0.02	4.54% - 42%
Lawsonella clevelandensis	6.46	0.0009	1.51% - 11%
Cutibacterium namnetense	23	0.009	0.0001% - 0.2%

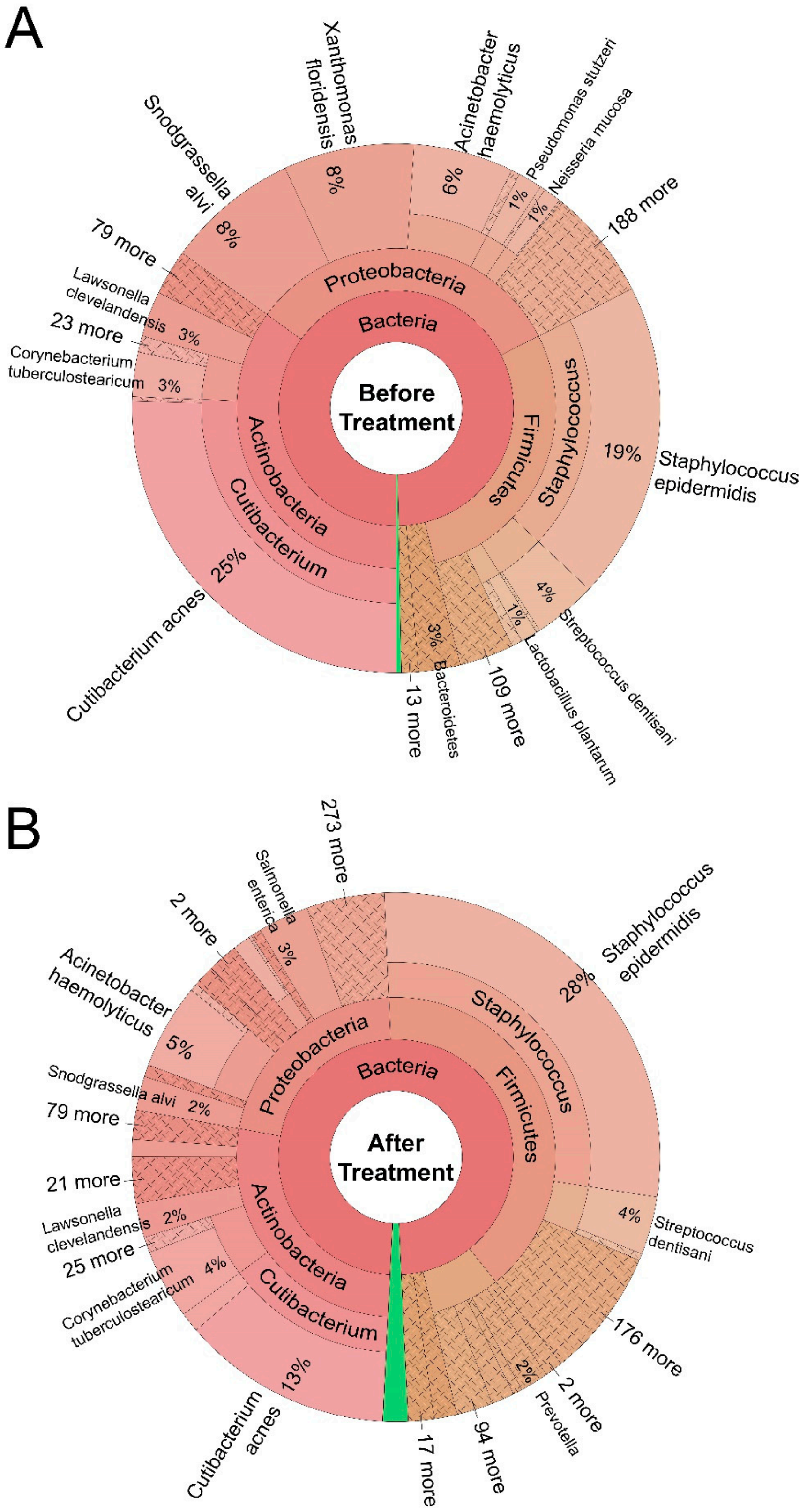

Figure S1. Krona graph on skin microbiota in acne patients (A) Before treatment, and (B) After 6 weeks of doxycycline.

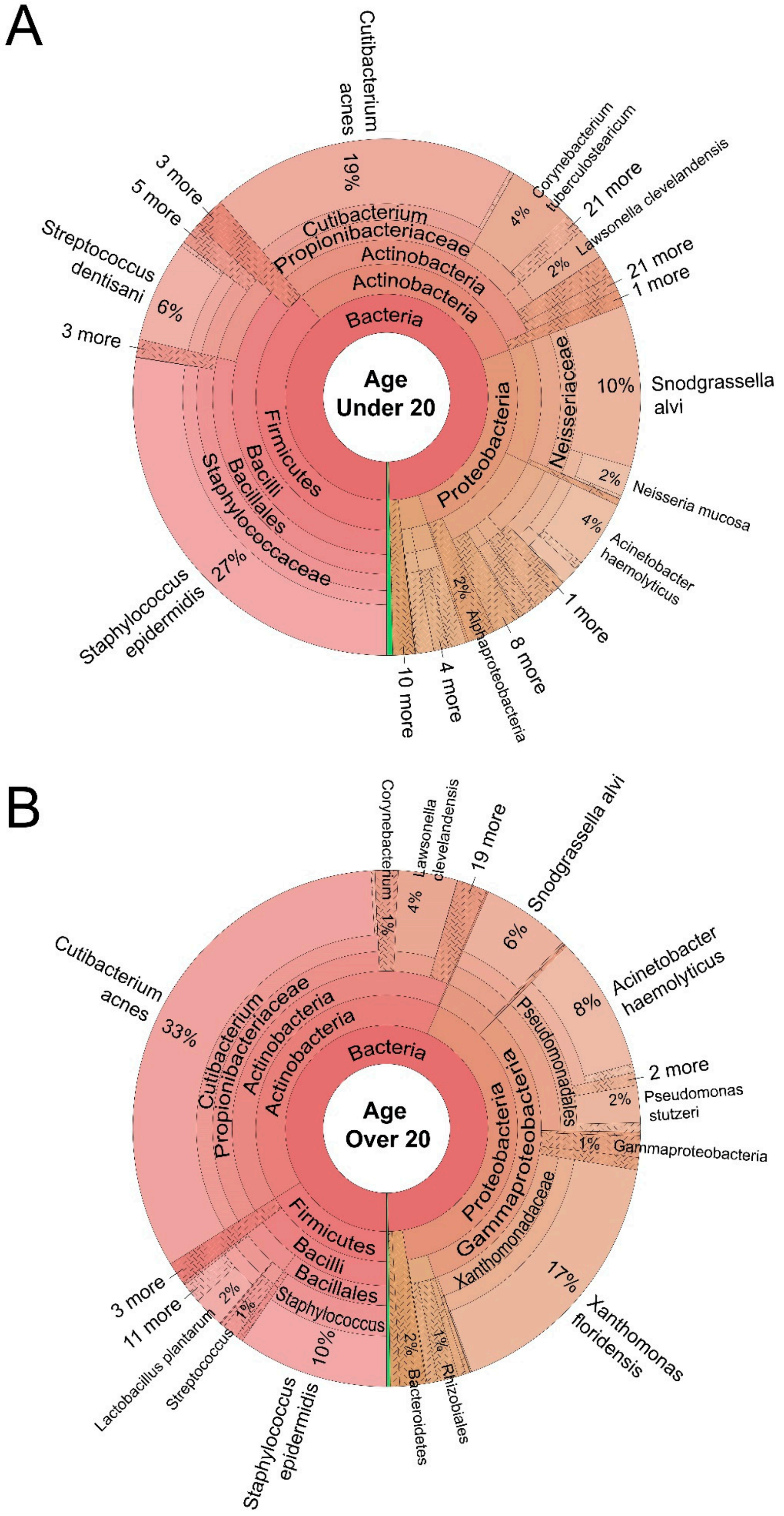

Figure S2. Krona graph on baseline skin microbiota in acne patients (A) Under 20, and (B) Over 20 years of age.

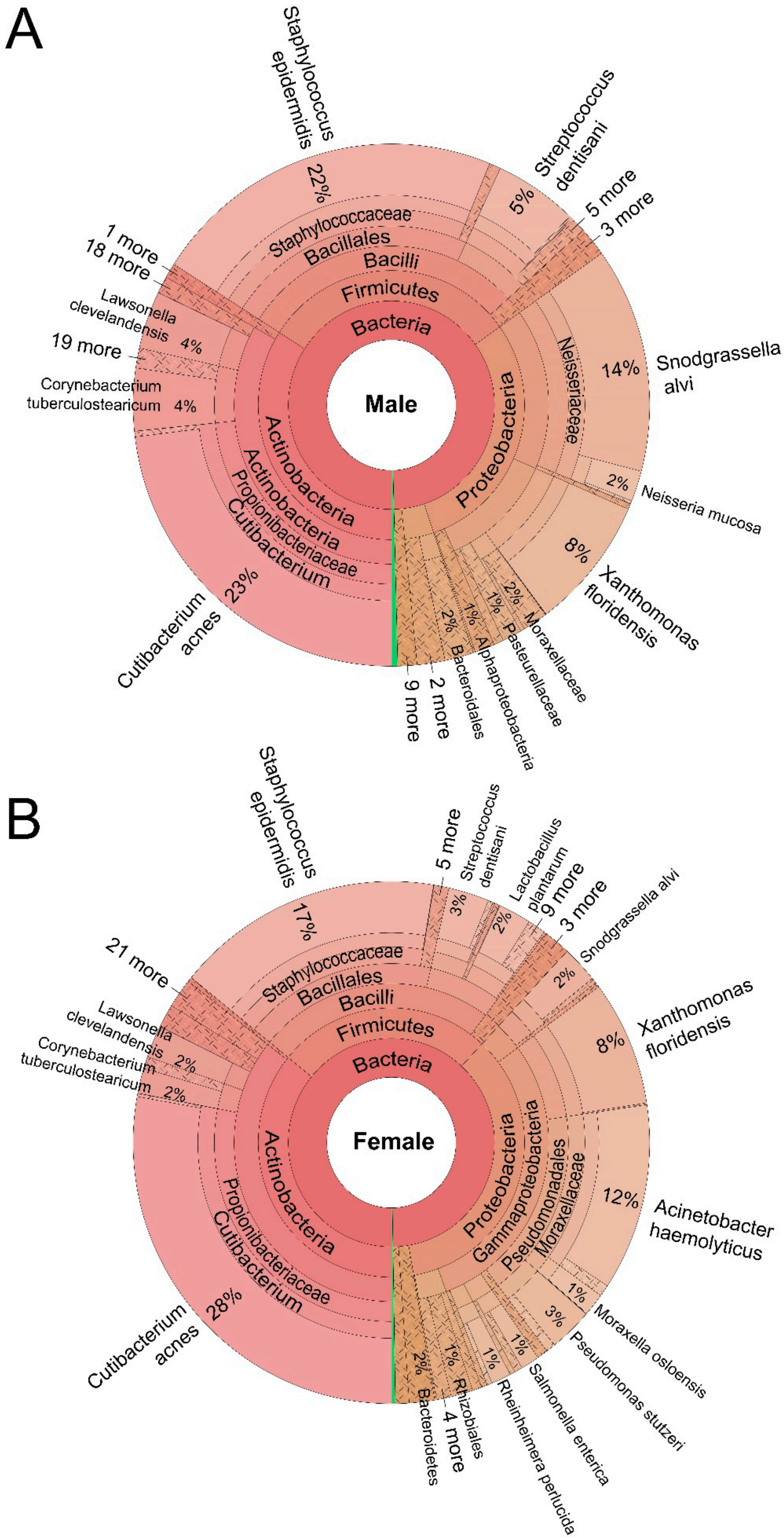

Figure S3. Krona graph on baseline skin microbiota in (A) Male, and (B) Female acne patients.

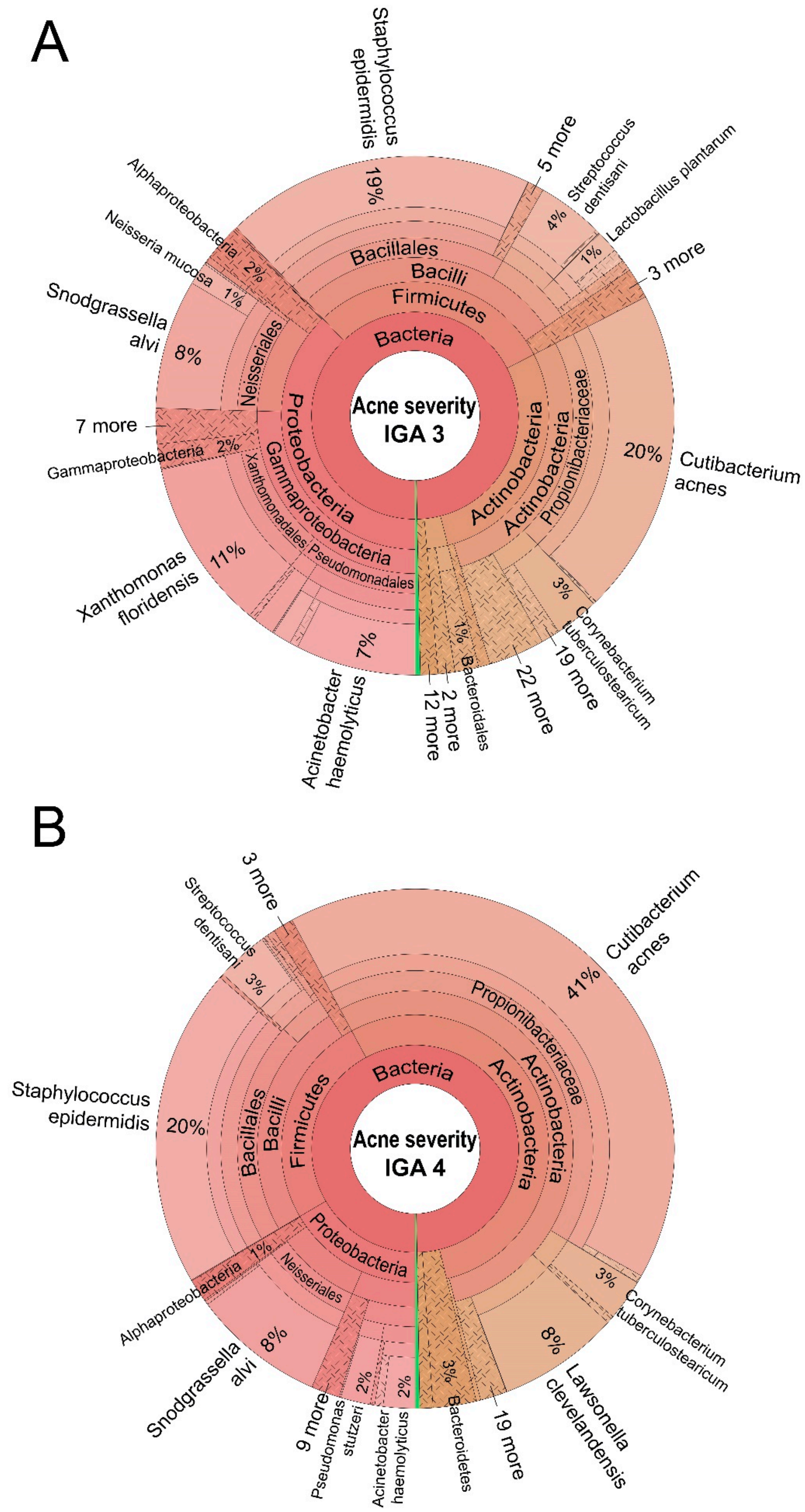

Figure S4. Krona graph on baseline skin microbiota according to acne severity. (A) IGA 3, and (B) IGA 4.
